# Pupil Diameter Changes after Anesthesia with Different Doses of Sufentanil under Ultrasound Monitoring

**DOI:** 10.1155/2022/6320973

**Published:** 2022-07-14

**Authors:** Xue-Lan Zhou, Li-Ji Xing, Hai-Rui Liu, Yu Qian, Jiang Zhu, Hong Xie

**Affiliations:** Department of Anesthesia, The Second Affiliated Hospital of Soochow University, Suzhou 215026, China

## Abstract

**Objective:**

This study aims to observe the changes in pupil diameter (PD) after anesthesia with different doses of sufentanil with the ultrasound method and observe whether pupil contraction is correlated with hemodynamic changes and bispectral index (BIS) values.

**Methods:**

A total of 124 patients between the ages of 18–65 with ASA I–II undergoing general anesthesia for surgery were enrolled in the study. According to the sufentanil dose initially injected, they were randomly divided into groups P, S1, S2, and S3, with 31 cases in each group. Group P was injected with normal saline. Group S1 was injected with 0.2 *μ*g/kg of sufentanil. Group S2 was injected with 0.4 *μ*g/kg of sufentanil. Group S3 was injected with 0.6 *μ*g/kg of sufentanil. Following propofol administration and eye closure, the pupil diameter (PD) of the patients in the four groups was observed and measured by ultrasound after the loss of consciousness (T1) and within 3 min after the sufentanil injection at an interval of 30 s (30 s (T2), 1 min (T3), 1 min 30 s (T4), 2 min (T5), 2 min 30 s (T6), and 3 min (T7)). PD, systolic blood pressure (SBP), diastolic blood pressure (DBP), heart rate (HR), and BIS values at T1–T7 were recorded.

**Results:**

The ultrasonic method was used to observe that different doses of sufentanil could make the patients' pupils contract. During anesthesia induction, the changes in PD have a positive correlation with SBP, DBP, HR, and BIS values.

**Conclusion:**

Ultrasound can become a new noninvasive method to monitor pupil changes during general anesthesia, and ultrasonic observation of pupil changes has great potential for individualized analgesia management in the perioperative period.

## 1. Background

Monitoring routine vital signs (respiration, blood pressure, ECG, pulse, and temperature) in the perioperative period and the depth of anesthesia has become more advanced, but pain monitoring during operations is still being explored [1]. After general anesthesia and loss of consciousness, the subjective numeric rating scale or visual analog scale cannot be used. Due to their limitations, some nociceptive stimulation monitoring methods have not been widely used in clinical practice. The pupil diameter (PD) of patients under general anesthesia does not depend on sympathetic system activation. When nociceptive stimulation mediated by A*δ* or C fibers happens, the Edinger–Westphal (E–W) nucleus in the dorsal midbrain is inhibited [2,3], and passive sphincter relaxation and pupil dilation will occur. It was found in subjects anesthetized with propofol or inhaled anesthetics that rapid and adequate pupil dilation was associated with the intensity of noxious stimulation, and compared with the vital signs, the pupil may be a more sensitive monitoring indicator for assessing nociceptive stimulus [4,5]. After using opioids, if the pupil is in the same dilation range, higher stimulus intensity is required [2]. These studies have proven that changes in PD can reflect the adequacy of analgesia in patients under general anesthesia in relation to nociceptive surgical stimulus. The stimulus is small in the anesthesia induction period, and the effect of opioid doses on balancing the hemodynamics and changes in PD is worth studying.

In recent years, the PD measurement method has become an emerging pain monitoring tool [6]. Clinical studies have shown that opioid analgesics can cause pupil constriction [7]. The electronic infrared pupilometer was applied in the PD measurement method reported in the literature, and multiple parameters can be obtained, including maximum and minimum PDs, the coefficient of pupil variation, the pupil incubation period for light reflection, and pupil contraction time. However, the electronic infrared pupilometer is usually equipped in special trauma centers, intensive care units, and eye wards, so there is no simple and accurate pupil measurement method. Ashot and other investigators found that B ultrasounds could quickly image the coronal pupil structure in real time with satisfactory and measurable images [8]. At present, studies regarding the application of PD in nociception monitoring increase daily, while there are no reports on the application of ultrasounds in measuring PD during induction of general anesthesia. In this study, the noninvasive ultrasonic pupil observation method was used for the first time to monitor the opioid effects in patients under general anesthesia. It was planned to observe the changes in PD caused by different doses of sufentanil in the anesthesia induction period, explore its correlation with the hemodynamic changes and the correlation between pupil changes and the depth of anesthesia, and investigate its feasibility as a new method for accurately quantifying dynamic pupil changes.

## 2. Materials and Methods

This study was a randomized, controlled, double-blind, prospective single-center study. The Ethics Committee of Soochow University (approval no.: JD-LK-2020-106–01) approved this study, and all patients signed informed consent. In this study, patients who underwent general anesthesia at the Second Affiliated Hospital of Soochow University from March 2021 to June 2021 were observed. Inclusion criteria: patients aged 18–65 years; with cardiac function Grade I–II; with ASA Grade I–II. Exclusion criteria: patients who underwent surgery under general anesthesia combined with intraspinal anesthesia or regional nerve blocks; body mass index (BMI) > 30 kg/cm^2^; patients who took sedative, analgesic, or psychotropic drugs; patients who took anticholinergic drugs before surgery; patients who took sympathetic or parasympathetic cardioactive drugs; patients who had eye disease or surgical histories, or bilateral pupils that were unequal; patients that had cranial neuropathy or arm mutilation. Withdrawal criteria: patients who had difficulty with mask ventilation or intubation during general anesthesia induction were excluded, and those who had to use sympathetic or parasympathetic cardiovascular drugs or atropine for various reasons during the observation period were rejected.

**Figure 1:**
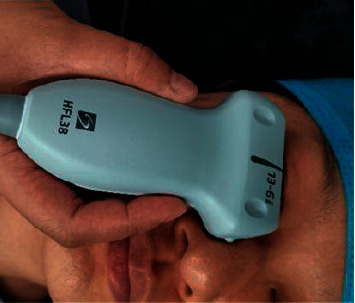
Probe position.

**Figure 2:**
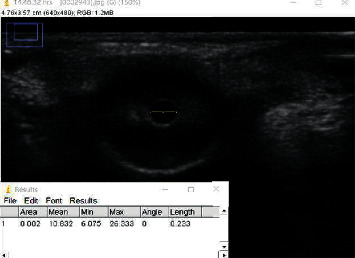
Pupil measurement with ImageJ software.

After entering the operating room, the patients were connected to the ECG, pulse oxygen saturation, noninvasive blood pressure, bispectral index (BIS), and muscle relaxation monitors. Intravenous access in an upper limb vein was secured and lactated Ringer's solution was infused at a rate of 7 ml/Kg/h. A photometer was used to measure ambient light intensity near each of the patient's eyes. The operating room was kept quiet, and patients were preoxygenated for 5 min. The propofol effect compartment concentration was set to 5 *μ*g/mL. After patients were unconscious, the rapid blood pressure measurement mode was enabled, and propofol was continuously pumped. The BIS was adjusted within the range of 40–60. The patients were divided into four groups according to the random number method and intravenously injected with different doses of sufentanil within 10 s. One minute after sufentanil administration, 0.6 mg/kg rocuronium was injected intravenously. According to the first dose of sufentanil injected, they were randomly divided into groups P, S1, S2, and S3, with 31 cases in each group. Group P was injected with normal saline. Groups S1, S2, and S3 were injected with sufentanil at a dose of 0.2 *μ*g/kg, 0.4 *μ*g/kg, and 0.6 *μ*g/kg for groups S1, S2, and S3, respectively. When the patients were in the state of eye closure, the linear array probe (frequency 6–13 MHz) of the portable two-dimensional ultrasonic instrument (EDGE) produced by Sonosite was placed under the lower eyelid of the right eye with minimum pressure (Figure 1). The probe was tilted close to the face, and the angle between the probe and the patient's face was adjusted to point toward the head and move slightly in that direction until satisfactory and complete coronal pupil and iris images were obtained. The PD of the patients in the four groups was observed after the loss of consciousness (T1) and within 3 min after sufentanil injection and measured by ultrasound at an interval of 30 s (30 s (T2), 1 min (T3), 1 min 30 s (T4), 2 min (T5), 2 min 30 s (T6), and 3 min (T7)). PD, systolic blood pressure (SBP), diastolic blood pressure (DBP), heart rate (HR), and BIS values at T1–T7 were recorded. After completing the observations, groups P, S1, and S2 were injected with sufentanil to 0.6 *μ*g/kg before endotracheal intubation. After image collection at T1–T7, ImageJ software was used to measure the horizontal left and right diameters of the pupils at each moment (Figure 2).

The SPSS 22.0 software was used for data analysis. The chi-square test was used for enumeration data. According to the data distribution, measurement data were expressed as mean ± standard deviation or median (range interquartile). A one-way ANOVA or Kruskal–Wallis H test was used to compare the differences in variables among the groups. Spearman's correlation analysis was used to analyze the relationship between PD and sufentanil doses. Pearson correlation analysis was used to analyze the relationship between PD, DBP, SBP, HR, and BIS. Two-factor repeated measure ANOVA was used to compare the PD among different groups and at different time points. *P* < 0.05 was considered statistically significant.

## 3. Results

### 3.1. General Data

In this study, 124 patients were included, and 10 were rejected. Finally, 114 patients were enrolled, including 29 cases in group P, 31 in S1, 27 in S2, and 27 in S3. The differences in age, gender, ASA grading, BMI, and intensity of light, as well as SBP, DBP, HR, BIS, and PD of patients in the four groups when awake after entering the operating room were not statistically significant (*P* > 0.05) (Table 1).

### 3.2. Pupil Diameter Changes in the Four Groups at T1–T7

**Figure 3:**
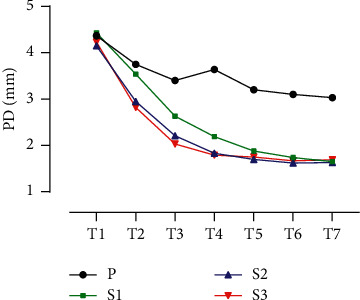
Comparison of pupil diameter among the four groups in the observation period.

Compared with group P, the PD of group S3 at T2–T7 decreased significantly, and the PD of groups S1 and S2 at T3–T7 decreased significantly, with statistical significance (*P* < 0.05). This showed that the PD of group S3 differed from group P at T2, while groups S1 and S2 started to differ at T3. In other words, when the sufentanil dose was 0.2 *μ*g/kg or 0.4 *μ*g/kg, the pupil contraction effect occurred in 60 s (at T3) after intravenous injection; when the sufentanil dose was 0.6 *μ*g/kg, the pupil contraction effect occurred in 30 s (at T2) after injection (see Table 2 and Figure 3).

In group P, compared with T1, the PD at T2–T7 decreased, with statistical significance (*P* < 0.05). Compared with T7, the PD at T1 and T4 increased, with statistical significance (*P* < 0.055). The difference in the PD at T2, T3, T5, and T6 was not statistically significant. This showed that in group P, the PD was kept at the same level at T2, T3, T5, and T6, in addition to the increase at T4 (see Table 2 and Figure 3).

In group S1, compared with T1, the PD at T2–T7 decreased, with statistical significance (*P* < 0.05). Compared with T7, the PD at T1–T6 increased, with statistical significance (*P* < 0.05). This showed that in group S1, the PD continued to decrease and reached the minimum at T7 without a plateau (see Table 2 and Figure 3).

In group S2, compared with T1, the PD at T2–T7 decreased, with statistical significance (*P* < 0.05). Compared with T7, the PD at T1–T4 increased, with statistical significance (*P* < 0.05). The difference in the PD at T5 and T6 was not statistically significant. This showed that in group S2, the pupil was contracted to its minimum at T5, and the period from T5 to T7 was the plateau of PD (see Table 2 and Figure 3).

In group S3, compared with T1, the PD at T2–T7 decreased, with statistical significance (*P* < 0.05). Compared with T7, the PD at T1–T3 increased, with statistical significance (*P* < 0.05). The difference in the PD at T4, T5, and T6 was not statistically significant. This showed that in group S3, the pupil was contracted to its minimum at T4, and the period from T4 to T7 was the plateau of PD (see Table 2 and Figure 3).

### 3.3. Correlation of Pupil Diameter with Systolic Blood Pressure, Diastolic Blood Pressure, Heart Rate, and Bispectral Index at T1–T7

**Figure 4:**
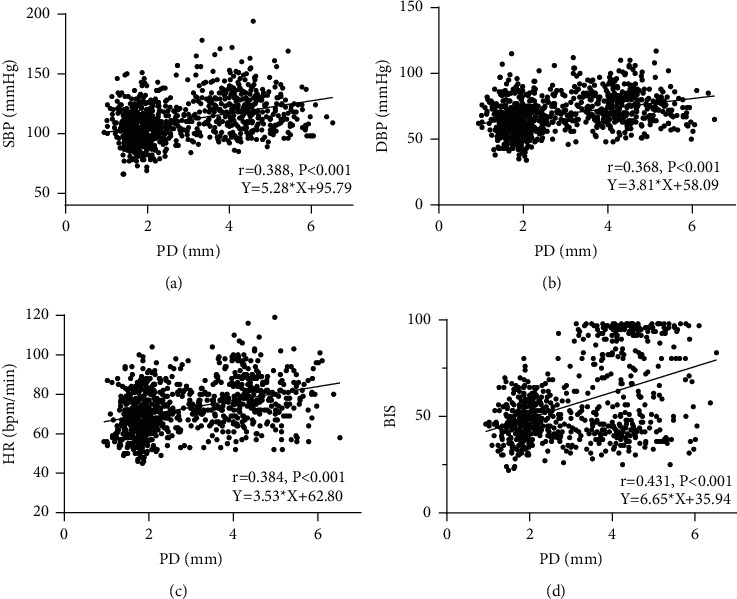
(a) PD and SBP correlation at T1–T7; (b) PD and DBP correlation at T1–T7; (c) PD and HR correlation at T1–T7; (d) PD and BIS correlation at T1–T7.

As shown in Figures 4(a)–4(d) and Table 3, Pearson correlation analysis and simple linear regression showed that PD was weakly correlated with SBP, DBP, and HR (*r* = 0.388, *P* < 0.001; *r* = 0.368, *P* < 0.001; *r* = 0.384, *P* < 0.001, respectively), and that PD had a moderate positive correlation with BIS (*r* = 0.431, *P* < 0.001).

## 4. Discussion

Propofol and opioids which are commonly used for induction of general anesthesia can affect pupil diameter [9,10]. Sabourdin et al. found a linear relation between PD and BIS in patients sedated with propofol alone. The main effect of propofol is to induce unconsciousness through inhibiting neurotransmission in the cerebral cortex, and there are also many subcortical effects. Propofol works on the PD through the subcortical structure (midbrain) [11]. The effect of propofol on pupil contraction is dose-dependent. In this study, the effect-site concentration of propofol was set to 5 *μ*g/mL for all four groups. Propofol was continuously pumped, and the BIS value was adjusted within 40–60. The level of sedation was kept consistent for as long as possible. The difference in PD among the four groups was caused by sufentanil alone (Figure 3).

Knaggs et al. studied the effects of intravenous injection of morphine (0.125 mg/kg), codeine (1 mg/kg), tramadol (1.25 mg/kg), and a placebo (10 mL 0.9% NaCl) on the pupils of 10 healthy volunteers. PD decreased by 26% after intravenous injection of morphine and codeine. PD did not decline until 150 min after tramadol administration. With drug metabolism, PD was recovered to the baseline value. The author considered that the changes in PD could reflect the pharmacokinetic characteristics of opioids, and measuring PD may play a role in monitoring the central effect of opioids [12]. Sufentanil acts on *μ* opioid receptors. With a strong lipophilic property, it can easily pass the blood-brain barrier. After intravenous administration, it works within 1–3 min and reaches its peak within 5–6 min. Thus, in this study, changes in PD were observed within 3 min (T1–T7) after intravenous injection of sufentanil. In group P, PD increased at T4 because patients often experienced injection pain and hand retraction reflection after the rocuronium injection at T3, and prior intravenous injections of lidocaine, opioids, and dexmedetomidine can significantly reduce the degree and incidence of local pain. Hence, rocuronium injection pain and PD increase did not occur in groups S1, S2, and S3, indirectly indicating that opioids block PD in response to noxious stimulation.

The effect of opioids on pupil contraction may be due to the effect on pupil retraction, but opioids may also directly act on the pupil sphincter because local morphine drips into the eyes and can also make pupils contract. Larson studied two brain-dead patients, and intravenous injections of fentanyl and morphine did not make their pupils contract, suggesting that opioid-induced pupil constriction must be mediated by the central nervous system. The exact site of action is unclear. It is generally believed that such an effect works by directly stimulating the preganglionic parasympathetic fibers in the midbrain E–W nucleus. This study showed that the sufentanil-induced pupil contraction speed was related to the dose. The larger the dose of sufentanil, the faster the pupil contraction. The minimum PD was reached at 120 s upon drug injection in group S3, while it took 180 s and 150 s for groups S1 and S2, respectively, to reach the minimum PD. Compared with group P, the PD of the other three groups decreased at 180 s, and there was no difference in the decrease range. Therefore, sufentanil-induced pupil contraction is “complete” or “none” during the induction period. PD alone cannot reflect the dose difference and analgesic effect of opioids.

Previous studies have shown that PD is a more sensitive measurement of noxious stimulation than the hemodynamic variables [4]. BIS monitoring may help in monitoring the depth of anesthesia, but not in titrating opioid dose [7]. Traditional judgment of depth of anesthesia is mainly based on routine indicators such as blood pressure, HR, and BIS, while PD is innervated by both sympathetic and parasympathetic nerves and reflects autonomic nerve balance. It was found in this study that PD showed a positive correlation with BIS, SBP, DBP, and HR at T1–T7 during the induction period (*P* < 0.001), but the correlation was not strong (Table 3), indicating that PD can reflect the change of patients from awakening to anesthesia like all vital signs. However, PD is influenced comprehensively by propofol and sufentanil, so it cannot be used to judge the depth of anesthesia accurately in the induction period.

## 5. Conclusion

Ultrasound can become a new noninvasive method to monitor the pupil changes of patients during general anesthesia, and ultrasonic observation of pupil changes has great potential for individualized analgesia management in the perioperative period. In subsequent studies, the pupillary intraoperative pain monitoring effect of ultrasound could be further explored by anesthesiologists to find whether it can be used to titrate the dose of opioids.

## Figures and Tables

**Table 1:** Comparison of demographics among the four groups.

	Group P (*n* = 29)	Group S1 (*n* = 31)	Group S2 (*n* = 27)	Group S3 (*n* = 27)	*P* value
Age (years)	48.6 ± 11.9	44.3 ± 10.4	45.2 ± 11.8	45.0 ± 14.5	0.546
Gender (male/female)	14/15	17/14	9/18	12/15	0.423
ASA grading (grade I/II)	22/7	25/6	20/7	20/7	0.924
BMI(kg/cm^2^)	24.1 ± 3.8	24.9 ± 3.1	23.1 ± 3.0	24.8 ± 3.0	0.154
Light intensity	120.7 ± 19.3	112.2 ± 21.7	113.0 ± 11.6	113.4 ± 15.7	0.238
Awake					
Systolic blood pressure (mmHg)	131.6 ± 14.3	134.2 ± 18.9	134.6 ± 19.7	132.4 ± 16.8	0.906
Diastolic blood pressure (mmHg)	83.2 ± 10.0	80.0 ± 11.1	80.1 ± 10.1	77.0 ± 11.2	0.208
Heart rate (bpm)	78.7 ± 13.9	76.7 ± 15.3	79.2 ± 12.7	81.3 ± 12.4	0.639
BIS value	93.9 ± 3.9	94.3 ± 5.3	94.0 ± 3.2	94.8 ± 3.9	0.848
Pupil diameter (mm)	4.38 ± 0.73	4.50 ± 0.76	4.41 ± 0.78	4.50 ± 0.60	0.887

**Table 2:** PD changes in the four groups at T1–T7 (‾*x* ± *S*).

Groups	T1	T2	T3	T4	T5	T6	T7
P	4.37 ± 0.80^#^	3.75 ± 1.00^*∗*^	3.40 ± 1.07^*∗*^	3.64 ± 1.07^*∗*^^#^	3.20 ± 1.21^*∗*^	3.10 ± 1.24^*∗*^	3.03 ± 1.35^*∗*^
S1	4.43 ± 0.88^#^	3.54 ± 1.01^*∗*^^#^	2.63 ± 0.87^a^^*∗*^^#^	2.19 ± 0.79^a^^*∗*^^#^	1.88 ± 0.44^a^^*∗*^^#^	1.74 ± 0.36^a^^*∗*^^#^	1.65 ± 0.31^a^^*∗*^
S2	4.15 ± 0.81^#^	2.95 ± 1.24^*∗*^^#^	2.21 ± 0.90^a^^*∗*^^#^	1.83 ± 0.38^a^^*∗*^^#^	1.70 ± 0.26^a^^*∗*^	1.62 ± 0.26^a^^*∗*^	1.63 ± 0.27^a^^*∗*^
S3	4.23 ± 0.73^#^	2.82 ± 1.06^a^^*∗*^^#^	2.03 ± 0.55^a^^*∗*^^#^	1.79 ± 0.23^a^^*∗*^	1.75 ± 0.26^a^^*∗*^	1.67 ± 0.22^a^^*∗*^	1.69 ± 0.22^a^^*∗*^

Compared with group P, ^a^*P* < 0.05; compared with T1, ^*∗*^*P* < 0.05; compared with T7, #*P* < 0.05.

**Table 3:** Correlation of PD with BIS, SBP, DBP, and HR at T1–T7.

Correlation	r value	*P* value
PD and BIS	0.431	<0.001
PD and SBP	0.388	<0.001
PD and DBP	0.368	<0.001
PD and HR	0.384	<0.001

## Data Availability

The datasets used and/or analyzed during the current study are available from the corresponding author on reasonable request.
